# IRF7 in the Australian Black Flying Fox, *Pteropus alecto*: Evidence for a Unique Expression Pattern and Functional Conservation

**DOI:** 10.1371/journal.pone.0103875

**Published:** 2014-08-06

**Authors:** Peng Zhou, Chris Cowled, Ashley Mansell, Paul Monaghan, Diane Green, Lijun Wu, Zhengli Shi, Lin-Fa Wang, Michelle L. Baker

**Affiliations:** 1 CSIRO, Australian Animal Health Laboratory, Geelong, Victoria, Australia; 2 Centre for Innate Immunity and Infectious Diseases, Monash Institute of Medical Research-Prince Henry Institute of Medical Research, Monash University, Clayton, Victoria, Australia; 3 Center for Emerging Infectious Diseases, Wuhan Institute of Virology, Chinese Academy of Sciences, Wuhan, China; 4 Program in Emerging Infectious Diseases, Duke-National University of Singapore Graduate Medical School, Singapore; Chang Gung University, Taiwan

## Abstract

As the only flying mammal, bats harbor a number of emerging and re-emerging viruses, many of which cause severe diseases in humans and other mammals yet result in no clinical symptoms in bats. As the master regulator of the interferon (IFN)-dependent immune response, IFN regulatory factor 7 (IRF7) plays a central role in innate antiviral immunity. To explore the role of bat IRF7 in the regulation of the IFN response, we performed sequence and functional analysis of IRF7 from the pteropid bat, *Pteropus alecto*. Our results demonstrate that bat IRF7 retains the ability to bind to MyD88 and activate the IFN response despite unique changes in the MyD88 binding domain. We also demonstrate that bat IRF7 has a unique expression pattern across both immune and non-immune related tissues and is inducible by double-strand RNA. The broad tissue distribution of IRF7 may provide bats with an enhanced ability to rapidly activate the IFN response in a wider range of tissues compared to other mammals. The importance of IRF7 in antiviral activity against the bat reovirus, Pulau virus was confirmed by siRNA knockdown of IRF7 in bat cells resulting in enhanced viral replication. Our results highlight the importance of IRF7 in innate antiviral immunity in bats.

## Introduction

Bats have been implicated in the spillover of many deadly viruses including rabies, henipaviruses (Hendra and Nipah), ebola virus, and the coronaviruses (CoV): severe acute respiratory syndrome (SARS-CoV) and the recently emerged Middle Eastern respiratory syndrome virus (MERS-CoV), all of which impose a significant threat to human health [1], [2], [3], [4], [5], [6]. As natural hosts, bats rarely show clinical signs of disease during infection [7]. How bats co-exist with viruses and the role of the bat innate immune system in controlling viral replication remain poorly understood [8]. Identifying the mechanisms responsible for controlling viral replication in bats has profound implications for the development of therapeutic strategies targeting viral infections in humans and other species.

One of the most important early anti-viral defenses in mammals is the IFN system, which not only provides pivotal protection immediately following infection but also shapes the adaptive immune response [9]. Of the three IFN families discovered, type I (including α and β) and type III (λ) IFNs respond directly to viral infection. Due to the importance of IFNs in controlling viral replication, the regulation of the IFN response has been extensively studied in humans and other mammals. Key to the regulation of IFN production and signaling is the IFN regulatory factor (IRF) transcription factor family. The IRF family consists of nine members which share functional and structural characteristics. However, only IRF1, IRF3, IRF5 and IRF7 have been implicated as positive regulators of type I IFN transcription, and only IRF3 and IRF7 are designated as antiviral IRFs [10],[11]. Since their first discovery within the biological context of Epstein-Barr virus latency, IRF7 was identified as the master regulator of the type I IFN-dependent immune response, and perhaps that of type III IFN as well [12],[13],[14].

IRF7 is expressed only at low levels in most cells but is constitutively expressed in certain immune cells such as plasmacytoid dendritic cells (pDC) which specialize in IFN production. Correspondingly, the tissue distribution of human IRF7 is restricted to immune tissues which contain large numbers of specialized immune cells including spleen, thymus and peripheral blood lymphocytes whereas non-immune tissues including the intestine and colon express almost undetectable levels of IRF7 [12]. Although IRF7 is expressed at low levels in most cell types, it is induced strongly by type I IFN mediated signaling in all cells [15]. Interestingly, multiple fish species (Japanese flounder, crucian carp, mandarin fish, snakehead fish and Atlantic salmon) have been demonstrated to express IRF7 constitutively in all tissues including both immune and non-immune tissues [16],[17],[18],[19],[20].

Viral sensing either by Toll like receptors (TLRs) or retinoic acid-inducible gene 1 (RIG-I)-like receptors can result in the activation of IRF7 and subsequent induction of IFNs [21],[22]. All TLRs with the exception of TLR3 activate IRF7 through the adaptor protein, MyD88 (myeloid differentiation primary response gene 88) through the MyD88-dependent pathway. MyD88 forms a complex with the kinases IRAK-4 (interleukin 1 receptor associated kinase 4), IRAK-1 and TRAF-6 (TNF receptor associated factor). This complex binds directly to IRF7 leading to ubiquitination by TRAF-6 and phosphorylation by IRAK1 or IKK-1 (IκB kinase-1) and translocation from the cytosol to the nucleus where IRF7 binds to promoter elements inducing IFN production [21],[23]. TLR3 and TLR4 activate IRF7 through the MyD88-independent pathway through the adaptor molecule TRIF (TIR-domain-containing adapter-inducing IFN-β) which forms a complex with TBK1 (TANK binding kinase 1), IKK-ε (inhibitor of nuclear factor-κb kinase e) and IRF7. In this case the phosphorylated IRF7 forms a homodimer or heterodimer with IRF3 and translocates to the nucleus where it binds to the IFN promoter via its DNA-binding domain to induce type I or type III IFN [24]. In IRF7 knockout mice, viral induced IFN production through the TLR3 (MyD88-independent) pathway is greatly impaired. As a result, mice become more susceptible to viral infection [13]. Although IRF3 has been reported to preferentially activate IFN-β over IFN-α genes, IRF7 is believed to efficiently activate both IFN-α and IFN-β [25]. The human IFN-β promoter region contains four positive regulatory domains (PRDs 1 to IV) that serve as binding sites for IRFs. In the human IFN-α promoter region there may be two or three PRD modules depending on the IFN-α subtype [26]. Due to the importance of IRF7 in the innate immune response, it is an active target for viruses to evade the host immune response [27]. A role for IRF7 in immunosurveillance has also been identified in breast cancer [28].

Using the Australian black flying fox (*P. alecto*) as a model species we have begun to explore the role of the IFN system in the control of viral replication in bats. We have demonstrated that TLRs, RIG-I-like receptors, and some IFN stimulated genes (PKR, Mx1 and OAS1) appear to be conserved in sequence compared to other mammals [29],[30],[31]. However, bats appear to have relatively higher expression of type III IFN and wider distribution of type III IFN receptors consistent with a role for type III IFNs in antiviral immunity [32],[33]. Bat genome analysis has also provided evidence for positive selection of genes within the IFN pathway, including TLR7, c-Rel, TBK-1, IFN-γ, ISG15 and RIG-I [34]. These changes may have occurred in response to the co-evolution of bats with viruses and may have consequences for the clearance of viral infections and the ability of bats to coexist with viruses. Due to the central role of IRF7 in the regulation of the IFN response, we performed sequence and functional analysis of *P. alecto* IRF7. Our results provide the first description of IRF7 in any species of bat and evidence for conserved IRF7 functional activity despite variation at the sequence level in the bat IRF7 gene.

## Materials and Methods

### Cells lines

All animal experiments were approved by the Australian Animal Health Laboratory (AAHL) animal ethics committee (protocol number 1389). Immortalized and cloned *P. alecto* kidney (PaKiT03) and lung (PaLuT02) cells established previously [35] were cultured in DMEM/F12-Hams (Sigma), supplemented with 10% foetal calf serum (FCS, Hyclone), 100 units/ml penicillin, 100 µg/ml streptomycin and 50 µg/ml gentamycin (Sigma). Human embryonic kidney HEK293T cells were cultured in DMEM supplemented with 10% FCS (Hyclone), 15 mM L-glutamine, 100 µg/ml penicillin, NEAA/Na-py/fungizone. All cells were maintained in a humidified atmosphere of 5% CO_2_ at 37°C.

### Viruses

Sendai virus (SeV, Cantell strain) was prepared in chicken embryos as described previously [29]. Pulau virus (PulV) was prepared and titered as described previously [33]. For infection of cells, virus was incubated with cells for one hour at 37°C, then replaced with normal cell culture medium for the indicated time.

### Genome and sequence analysis

Full-length IRF7, IRF3 and MyD88 open reading frames (ORFs) were identified in the *P. alecto* genome (NCBI ID *P. alecto* ASM32557v1) [34] using BLASTX. For comparative purposes, sequences were obtained from the current genome assemblies from the Ensembl database for the following species: ENSP00000380697, *Homo sapiens* (human); ENSMUSP00000095565, *Mus musculus* (mouse); ENSECAP00000007698, *Equus caballus* (horse); ENSSSCP00000013664, *Sus scrofa* (pig); ENSBTAP00000056564, *Bos taurus* (cow). The microbat *Myotis davidii* IRF7 amino acid sequence was deduced from IRF7 ORF annotated from the published *M. davidii* genome (NCBI ID *M. davidii* ASM32734v1) [34]. The *P. alecto* IRF7 sequence has been submitted to GenBank under accession number KJ534586.

Sequence alignment was performed using ClustalX and visualized using GeneDoc (http://www.nrbsc.org/gfx/genedoc/index.html). Alignment files were visualized using EMBOSS Plotcon to determine the conservation of IRF7 proteins among different species. Genomic intron-exon maps of the genes were drawn using Fancy Gene v1.4 by comparing individual IRF7 ORFs of *P. alecto*, horse and human (http://host13.bioinfo3.ifom-ieocampus.it/fancygene/). Phylogenetic trees were constructed using the neighbour joining method and MEGA4.1 program with 1000 bootstrap replicates [36].

### Plasmid constructs

Primers listed in Table 1 were designed based on the *P. alecto* genomic sequences and used in RT-PCR to amplify IRF7, IRF3 and MyD88 from RNA extracted from freshly isolated bat splenocytes. To construct expression plasmids, PCR products corresponding to full-length IRF3 and IRF7 were ligated directly to Vivid Colors pcDNA 6.2/EmGFP TOPO vector (Life Technologies) with an N-terminal GFP tag. The MyD88 ORF was ligated to the pFLAG-CMV2 expression vector (Sigma) using restriction enzymes *Not*I and *Sal*I with an N-terminal FLAG tag for detection. To generate a truncated bat IRF7 (tIRF7) that lacked the MyD88 binding region at amino acids (aa) 234–298, overlapping PCR was performed [37]. The resulting tIRF7 PCR product was ligated to the pcDNA 6.2/EmGFP TOPO vector. The human IRF7 (hu-IRF7) and hu-MyD88 plasmids have been described previously [38]. Hu-IRF7 is in the pEGFP-N1 vector and hu-MyD88 is in the pEF-Bos vector with an N-terminal FLAG tag.

**Table 1:** 

Gene	primer	sequence 5′-3′	Application
IRF7	IRF7-1F	ATGGCCGCCGCCCGC	Full-length ORF amplification
	IRF7-1R	CTAGGCGGGCTGCTCC	Full-length ORF amplification
	IRF7-2F	CGACCCGCATAAAGTGTATGAG	Real-time PCR
	IRF7-2R	CCTGGTTAATGCCTGGACTTTC	Real-time PCR
	siIRF7-1	GCGCATACCTGGAGAGCGT	siRNA for IRF7
	siIRF7-2	CGTCATGCTGCACGACAAT	siRNA for IRF7
	siIRF7-3	GGAAGCACTTTTCGCGGAA	siRNA for IRF7
	siIRF7-4	CCGCGAAAGTGCACTCCGA	siRNA for IRF7
IRF3	IRF3-1F	ATGGCTACCCCAAAGCCG	Full-length ORF amplification
	IRF3-1R	CTAGAAATCCATGTCCTCGACCAG	Full-length ORF amplification
	IRF3-2F	TCGACCTGAAGCCCTTCGT	Real-time PCR
	IRF3-2R	GGCGAGCGTCCACTTCCT	Real-time PCR
MyD88	MyD88-1F	TAAGCGGCCGCGACCATGGCGGCACAAGTTC	Full-length ORF amplification
	MyD88-1R	GAGGTCGACTCAGGGCAAGGACAGG	Full-length ORF amplification
IFN-βP	IFN-βP-1F	GTCGGTACCGTAATTGAAAAATAAATCTG	IFN-β promoter construction
	IFN-βP-1R	TGCAAGCTTCGTTGGCAATGTGAATGTC	IFN-β promoter construction

Mouse IFN-α4, IFN- α6 and human IFN-β promoter plasmids, abbreviated as Mu_IFN- α4P, -α6P and Hu_IFN- βP respectively, have been described previously [21]. The bat IFN-β promoter plasmid was constructed using sequence 1000 bp upstream from the start codon of IFN-β ORF from the *P. alecto* genome. Promoter prediction was performed using the online transcriptional start site prediction tool, Matinspector in the Genomatix software suite (http://www.genomatix.de/cgi-bin//matinspector_prof). Regions containing putative IRF3 or IRF7 binding sites were identified from −221 to −70 bp from the ATG of the bat IFN-β gene by comparison with human IFN promoters and cloned into the pGL4.1 expression vector (Promega). A transfection control pRL-Tk plasmid containing Renilla luciferase was obtained from Promega. Details of primers used during plasmid construction can be found in Table 1.

### Luciferase promoter assay

HEK293T cells were transfected using Fugene 6 (Promega) according to the manufacturer’s instructions. Approximately 2×10^5^ cells per well in a 24-well plate were co-transfected with 100 ng of the relative IFN promoter plasmids, 50 ng of pRL-TK (Promega) served as an internal control. Where indicated, expression plasmids for bat IRF7, human IRF7 or bat MyD88 were included in the transfection mix. Cells were harvested 30 h post-transfection and lysed using passive lysis buffer provided in the following kit. Luciferase activities were determined using the dual-luciferase assay system (Promega) using a Thermo Fluoroskan ascent FL machine. For promoter experiments in PaKiT03 cells, similar transfections were performed using lipofectamine 2000 (Life Technologies).

### IRF7 knockdown

A smartpool consisting of four small interfering RNAs (siRNAs) targeting bat IRF7 (siIRF7) was designed based on the full-length IRF7 ORF sequence using Dharmacon custom services (Thermo). Information of the four siIRF7 can be found in Table 1. Transfection of siIRF7 was performed in PaKiT03 cells using the Neon transfection system (Life Technologies) according to the manufacturer’s instructions. Briefly, 20 nM of each of the four siIRF7 was used for every 10^5^ cells. Cells were harvested into RLT lysis buffer (RNeasy kit, Qiagen) 48 h post-transfection and stored at −80°C prior to RNA extraction.

### Quantitative reverse transcription PCR (qRT-PCR)

RNA was extracted from cell lysate from the siRNA knockdown experiment using a Qiagen RNeasy kit and converted to cDNA using the Quantitect reverse transcription kit for real-time PCR (Qiagen). All experiments were performed according to manufacturer’s protocols. Preparation of cDNA from 12 *P. alecto* tissues including brain, kidney, liver, lung, lymph nodes, spleen, heart, small intestine, wing, salivary gland, thymus and testis from three individual bats and from polyI:C stimulated PaLuT02 cells has been described previously [33]. For each sample, 1 µg RNA was applied to reverse transcription using the Quantitech reverse transcription kit (Qiagen). IRF7 qRT-PCR primers were designed using Primer Express 3.0 (Applied Biosystems) with default parameter settings and are listed in Table 1. Primers for IFN-β and 18s rRNA have been described previously [33]. Reactions were carried out using EXPRESS SYBR GreenER qPCR Supermix Universal (Life technologies) in an Applied Biosystems 7500 Fast Real-Time qRT-PCR instrument. For each reaction from cDNA, 2 µl of 1∶5 diluted cDNA were used with a final concentration of 200 nmol of each primer. The cycling profile for cDNA samples consisted of an initial denaturation at 94°C for 2 minutes followed by 40 cycles of 94°C for 15 seconds, 60°C for 1 minute, followed by melt curve analysis. Expression levels of target genes were calculated using the standard curve method after normalisation to the housekeeping gene 18s rRNA.

### Indirect immunofluorescence assay (IFA) confocal microscopy

PaKiT03 cells were seeded onto glass coverslips in 24-well plates at 10^5^ cells per well one day before transfection. They were transfected with 200 ng each of GFP-IRF7 and FLAG-tagged MyD88 (human or bat) using Lipofectamine 2000 (Life Technologies). At 16 h post-transfection, cells were fixed in 4% paraformaldehyde in PBSA at room temperature for 40 minutes. After removal of fixative, cells were washed three times with PBSA, followed by treatment with 0.1% Triton X-100 for 10 minutes and blocked with 0.5% BSA in PBSA for 30 minutes. Mouse anti-human MyD88 antibody (Santa Cruz, CAT. sc11356) was diluted 1∶1000 in 0.5% BSA and applied to cells and incubated at 37°C for 1 h. Cells were then incubated with Alexa 488 conjugated goat anti-mouse secondary antibody at 37°C for 1 h and washed three times in PBSA. Nuclei were labelled with DAPI and coverslips were mounted on glass slides for analysis. All slides were examined under a Leica confocal microscope (Leica, Germany).

### Immunoprecipitation analysis

The immunoprecipitation method for analysis of IRF7 and MyD88 has been described previously [38]. In brief, 2×10^6^ HEK293T cells were seeded 24 h prior to co-transfection with 1.25 µg of both FLAG-MyD88 and GFP-IRF7 (human or bat) using Fugene 6 (Promega). Cells were then lysed 24 h later with lysis buffer (50 mM Tris (pH7.4), 1.0% Triton X-100, 150 mM NaCl, 1 mM EDTA, 2 mM Na_3_VO_4_, 10 mM NaF, 1 mM PMSF and protease cocktail inhibitor mixture; Promega). Cell lysates were cleared by centrifugation (9000 g, 10 min, 4°C), and then pre-cleared with protein G-Sepharose beads for 30 min at 4°C and FLAG-MyD88 immune complexes were immune-precipitated from supernatant using anti-FLAG M2-agarose (Sigma) conjugated beads for 2 h at 4°C. Beads were washed with 3 X lysis buffer and eluted by boiling beads in 5 volumes of SDS PAGE sample buffer. Lastly, SDS PAGE and western blot analysis were performed using the samples obtained. For the blotting, anti-human MyD88 rabbit antibody and anti-human IRF7 goat antibody (both from Santa Cruz) were used at 1∶1000 dilution during primary antibody incubation and AP-conjugated goat anti-rabbit or rabbit anti-goat secondary antibody (both from Life technologies) were used at 1∶2000 dilution.

## Results

### Characterization of *P. alecto* IRF7

To explore possible differences in the bat antiviral immune system which may influence the association between bats and viruses, IRF family members were chosen as primary targets due to their importance in IFN induction and signaling [10]. All IRF members from IRF1 to IRF9 were identified in the bat genome, indicating their relative conservation at the family level. We next performed sequence analysis on the four known positive regulators of type I IFN transcription in humans; IRF1, IRF3, IRF5 and IRF7. Comparison of the sequence similarity of the deduced protein sequence of human and bat IRFs demonstrated significant conservation of IRF1 (96%), IRF3 (88%) and IRF5 (87%). In contrast, bat IRF7 shares only 64% and 55% amino acid similarity to human and mouse respectively. To determine whether the relatively low sequence conservation of the bat IRF7 gene compared to human and mouse affects the functional activity of bat IRF7, this gene was chosen for further functional analysis.

**Figure 1:**
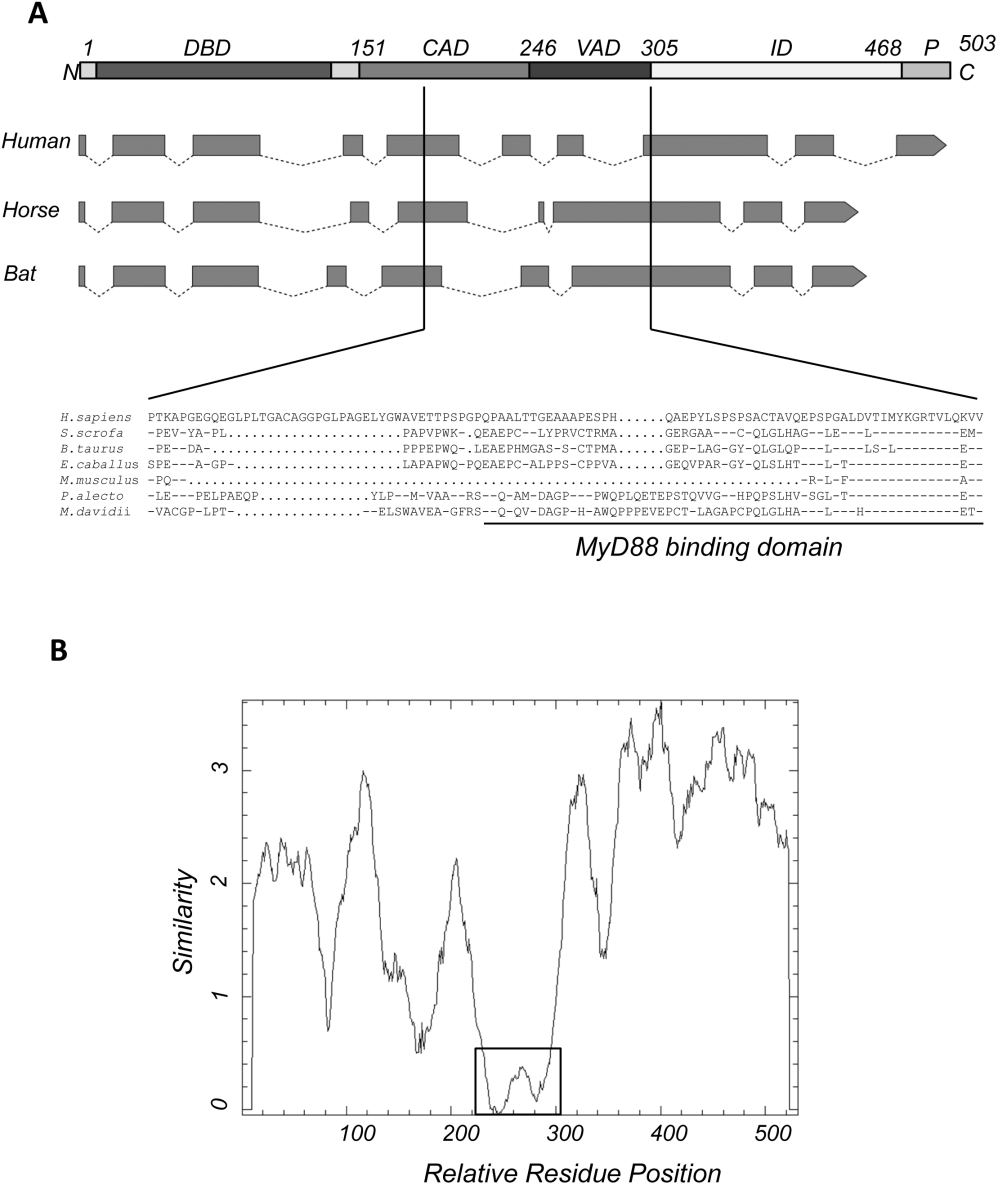
Comparison of bat IRF7 with IRF7 from other species. (A) Functional domains of IRF7 were drawn (top) based on human IRF7 [37]. The intron-exon structure of IRF7 from human, horse and bat were predicted by alignment of individual ORFs to their respective genomes. Solid boxes indicate exons while dotted lines represent introns; arrows mark the direction and the end of the genome structure. The deduced protein sequence of the region corresponding to human MyD88 binding motif (aa 247–305) from seven species including human, pig, cow, mouse, horse, *P. alecto* bat and *M. davidii* bat was aligned. DBD: DNA binding domain; CAD: constitutive activation domain; VAD: virus-activated domain; ID: autoinhibitory domain; P: phosphorylation sites, serine-rich domain. Dashes indicate similarity; dots indicate gaps. (B) Sequence similarity plot of *P. alecto* IRF7 with IRF7 from human, mouse, cow, pig, horse and *M. davidii* using a 20 amino acid sliding window and created using EMBOSS Plotcon (http://emboss.bioinformatics.nl/cgi-bin/emboss/plotcon). The similarity across the whole ORF between these species is shown by scores. The lower the score the lower the sequence conservation. The least conserved region is boxed.

The recently published *P. alecto* whole genome sequence and transcriptome data were used to identify the IRF7 gene [39],[40]. Primers based on the genomic IRF7 sequence were used to amplify full-length IRF7 by PCR from bat spleen cDNA resulting in the identification of a single full length IRF7 transcript. By aligning the bat IRF7 cDNA sequence with the corresponding region in the *P. alecto* genome, we were able to determine the intron and exon structure of this gene. As shown in Figure 1, bat and horse IRF7 have nine exons compared to the ten exon structure of human IRF7. Horse was included in this comparison due to the close phylogenetic relationship between bats and horses (figure S1 and [34]). Nine exons were also identified in IRF7 genes from other Laurasiatheria species, including pig, cow and dog. Of the sequences available in the Ensembl database, the ten exon structure appears to be typical only among primate IRF7 genes (data not shown).

Analysis of the putative bat IRF7 promoter region around 1000 bp upstream of the start site of the ORF resulted in the identification of two IFN stimulated response elements (ISREs) and one nuclear factor kappa B (NF-κB) binding site. To determine whether the presence of two ISRE sites was unique to bats, the IRF7 promoters of other species (human, mouse, horse, cow, dog, cat and rat) were also examined using the publicly available databases. All species examined contain two ISRE sites (one ISRE and one IRF binding site) with the exception of human which has a single ISRE (data not shown).

Based on the deduced protein sequence, *P. alecto* IRF7 was aligned with six other species: human, mouse, pig, cow, horse and the microbat, David’s myotis (*M. davidii*) available from the recently completed genome sequence [40]. These species were chosen because human and mouse have been well studied, while all other species are phylogenetically close to *P. alecto* [34]. Full-length IRF7 contained multiple functional domains including an N-terminal DNA-binding domain (DBD), followed by a constitutive activation domain (CAD), virus-activated domain (VAD), inhibitory domain (ID), and C-terminal serine-rich region (Figure 1; [22],[37]. The VAD is responsible for binding to the upstream activators of IRF7: MyD88, TRAF6 or TBK-1, while the serine-rich region is the target for virus-inducible phosphorylation [21],[41]. From the alignment, bat IRF7 appears to have a conserved DBD (Figure S2), C-terminal serine-rich domain which is the target of phosphorylation, and auto-inhibitory domain [37]. However, the region between amino acids 200–300, which corresponds to the VAD and contains the MyD88 binding site is not conserved in sequence among all species (Figure 1B). Furthermore, in the putative MyD88 binding region, both bats are less conserved not only to human but also to other Laurasiatheria species shown here (pig, cow and horse). As the MyD88-IRF7 pathway is critical to ssRNA-induced human IFN production, alignment was performed between bat MyD88 and MyD88 from human, mouse, pig, cow and horse. No significant change that would potentially alter the functionality of bat MyD88 was identified based on sequence analysis (figure S3).

### *P. alecto* IRF7 has a broad tissue distribution and is further induced following stimulation

**Figure 2:**
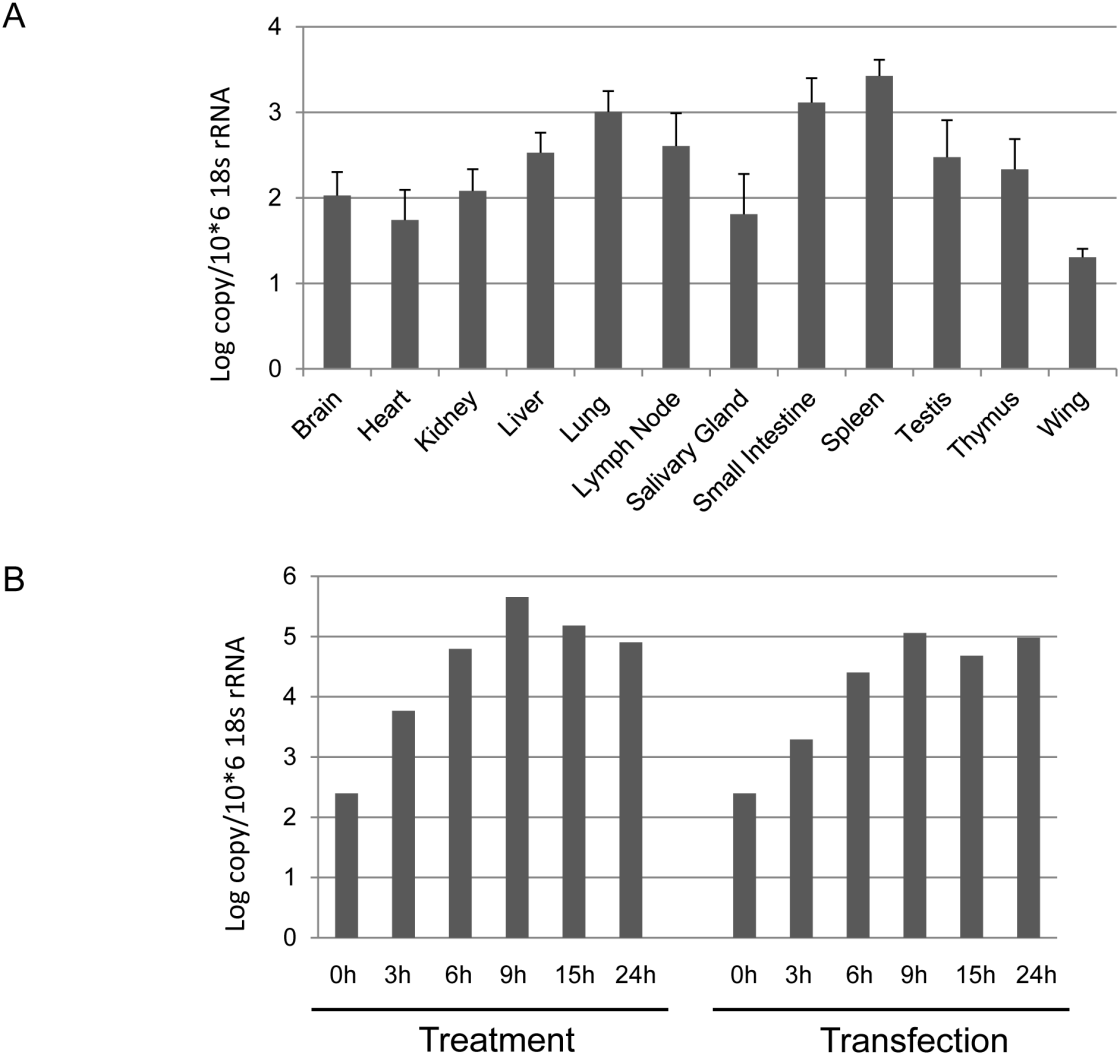
qRT-PCR detection of bat IRF7 (A) mRNA expression in *P. alecto* tissues. Data were normalized with the housekeeping gene 18s rRNA and represent mean results from three individual apparently-healthy wild-caught bats. Error bars represent standard errors (B) Production time course of bat IRF7 following polyI:C stimulation in the bat lung PaLuT02 cell line. Cells were either treated with polyI:C or mixed with Lipofectamine 2000 (transfection) with polyI:C and collected at the indicated time points for RNA extraction and qRT-PCR analysis. The data were normalized against the 18s rRNA gene.

To determine the tissue distribution of bat IRF7, its transcription was examined in a range of immune and non-immune associated bat tissues. The tissues were from three apparently healthy bats caught from the wild in which the IRF7 level should represent the normal expression pattern in wild bats. As shown in Figure 2A, bat IRF7 is widely expressed among all bat organs at the mRNA level, with spleen, small intestine and lung having the highest IRF7 expression and wing and salivary gland the lowest. With the exception of the wing, all other tissues showed similar expression levels of IRF7 with around 10-fold difference between spleen which had the highest expression and salivary gland with the lowest. This pattern differs from the transcription pattern of bat TLR7, 8 and 9, which appear to be predominantly expressed in immune tissues [30].

Next, the inducibility of IRF7 by a known virus mimic was explored by testing IRF7 transcription using our cloned and immortalized bat lung cell line (PaLuT02 cells) following stimulation with the double stranded RNA (dsRNA) ligand, polyI:C. The PaLu02 cell line has previously been demonstrated to produce IFN in response to polyI:C in a dose dependent manner [42]. As shown in Figure 2B, stimulation by either treatment or transfection with polyI:C, which mimics IFN production through TLR3 or RLH pathways respectively resulted in strong induction of IRF7. Notably, IRF7 mRNA was induced at the earliest time point of 3 h following stimulation, to a peak level of around 1000 times higher than mock treated cells, highlighting the importance of IRF7 in early antiviral defense in bats. These data are consistent with bat IRF7 being inducible by both TLR and RLH pathways. In summary, bat IRF7 is constitutively transcribed in all tissues and cell lines tested (PaLuT02 cells and PakiT03 cells, below) and can be strongly induced by dsRNA. Due to the limitations of the human IRF7 antibody used in this study, the expression of IRF7 at the protein level awaits the development of a suitable bat specific reagent.

### Bat IRF7 induces IFN in a dose-dependent manner

**Figure 3:**
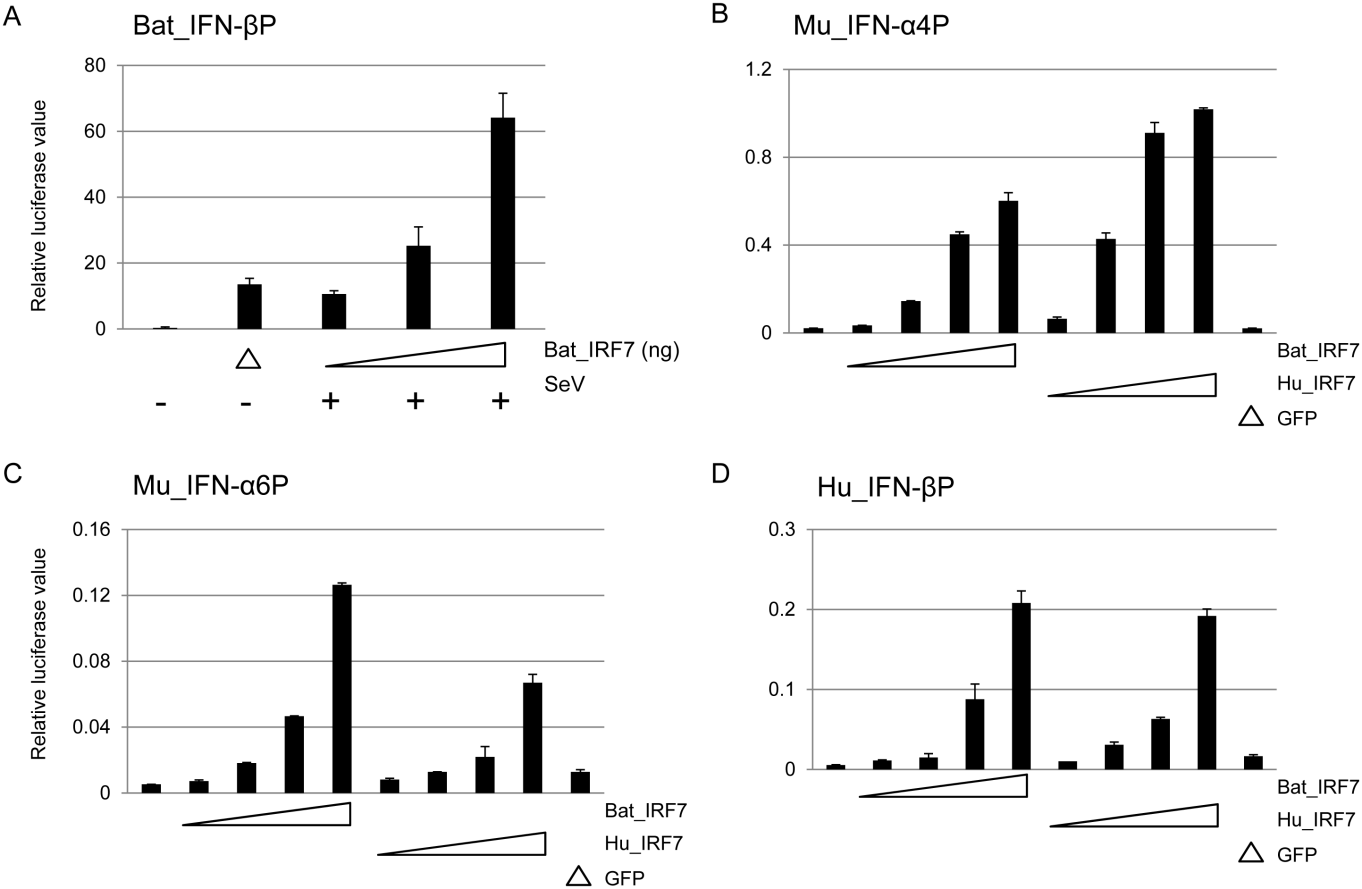
IFN-β is induced in a dose-dependent manner by bat IRF7. (A) Sendai virus (SeV) induced higher IFN-β induction with an increasing dose of bat IRF7. Bat PaKiT03 cells (2×10^5^ per well) were cotransfected with 0, 10, 50 or 100 ng pCAGGS-bat IRF7 (left to right, last three bars) and bat IFN-β promoter plasmid. 24 h post-transfection, cells were infected with 100 HAU SeV per well or mock infected (left to right, first two bars, Δ indicates transfection with 100 ng pCAGGS-bat IRF7) followed by promoter activation assay six hours after infection. Values show the mean of two experiments and error bars indicate standard errors. (B–D) HEK293T cells were transiently transfected with 0, 1, 10, 100 or 500 ng of expression plasmids for either bat (left to right, first five bars) or human IRF7-GFP (next four bars) with mouse IFN-α4 (B) IFN-α6 (C) or human IFN-β (D) promoter plasmid. GFP expression plasmid was used at 500 ng per well as negative control. After 30 h, cells were analysed for promoter activity by reporter gene assay. Similar results were obtained in two independent experiments. Values show the average of two experiments and error bars indicate standard errors.

To test the ability of IRF7 to induce IFN-β production a bat IFN-β promoter assay was used. The putative promoter region of the bat IFN-β gene was examined and predicted to contain IRF7 and IRF3 binding modules (figure S4). This region was ligated to the pGL4.1 luciferase reporter vector. Interestingly, the bat IFN-β promoter contains one residue difference in its PRDI which may abolish its ability to bind to IRF3 or IRF7 [43]. In contrast, the second PRD (PRDIII) is almost identical to the corresponding domain of the human IFN-β promoter region and was therefore predicted to be functional [44]. Comparison of the PRDI domain of other closely related species available in the Ensembl database and the microbat, *M. davidii*, revealed the disruption of PRDI is unique to *P. alecto* (Figure S4). In the bat immortalized kidney cell line (PaKiT03 cells), a plasmid encoding bat IRF7 was co-transfected with the bat IFN-β promoter plasmid for 24 h and cells were then infected with SeV for another 6 h before performing the luciferase test. The PaKiT03 cells were chosen for these experiments due to their ability to be successfully transfected and SeV was chosen due to its potent ability to induce IFNs and other cytokines in the absence of viral products that block the IFN response [45]. Results clearly indicate that IRF7 alone can induce IFN-β activation and SeV induces enhanced activation of IRF7 in a dose dependent manner (Figure 3A).

To examine whether the poorly conserved MyD88-binding region in bat IRF7 influenced its transactivation potential, bat IRF7 was compared with that of human IRF7 using IFN promoter assays. This experiment was designed to test the hypothesis that the sequence differences identified in the bat IRF7 region do not affect its ability to activate IFN transcription. This response was tested using mouse IFN-α4, mouse IFN-α6 or human IFN-β promoter plasmids co-transfected with increasing doses of bat or human IRF7 expression plasmids in HEK293T cells. As shown in Figures 3B–D, bat IRF7 activates all three promoters in a dose-dependent manner. Together with the results described above using the bat IFN promoter, these results demonstrate that bat IRF7 is capable of activating IFN in bat cells following stimulation with SeV or in human cells co-transfected with human or mouse IFN promoters. Cells transfected with mock or empty vector failed to activate the IFN promoters significantly. Although identical doses of human and bat IRF7 plasmid were used in these experiments, the results are not statistically comparable due to the difference in IRF7 protein expression from the two plasmids. Of note, we used human HEK293T cells and mouse IFN-α and human IFN-β promoters in this assay because they are a well established system for testing the activation of IFN [21]. Given the high conservation in the DNA-binding domain of bat IRF7, it was predicted to be capable of binding to the human and mouse IFN promoters followed by activation by downstream factors.

### siRNA knockdown of bat IRF7 reduces IFN production and increases viral replication

**Figure 4:**
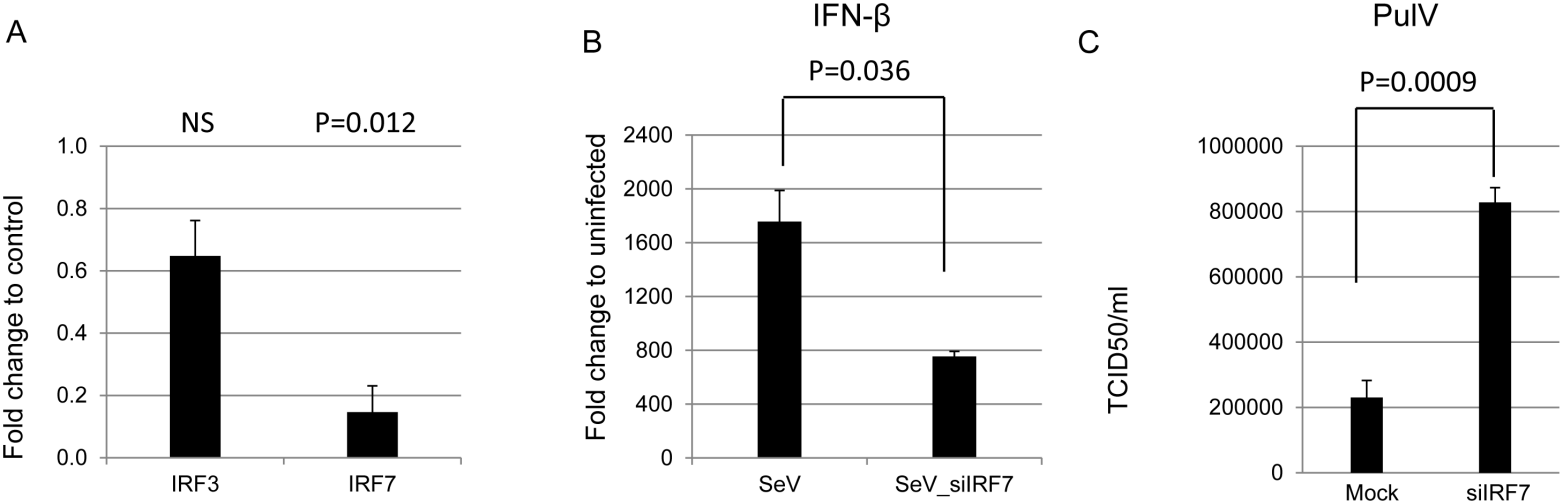
siRNA knockdown of bat IRF7 reduces IFN production and increases viral replication. (A) siRNA knock down of bat IRF7 in bat PaKiT03 cells. Cells were transfected with a final concentration of 20 nM of siIRF7 and collected 24 hrs later for qRT-PCR analysis of IFN-β mRNA. Knock down of bat IRF7 mRNA was compared with IRF3 which served as an indication of off-target effects. (B) Knockdown of bat IRF7 significantly reduced IFN-β mRNA induction by SeV. Cells were transfected with 20 nM siIRF7 for 24 h followed by infection with SeV for a further 6 h and then collected for qRT-PCR analysis. IFN-β mRNA following SeV or SeV plus siIRF7 was measured. Results represent the mean of triplicate samples. Error bar represent the standard error. The p-values were determined relative to control untransfected cells using two sample t-tests assuming unequal variances. NS, not significant. (C) Bat PulV grows to a high titre in bat IRF7-silenced PaKiT03 cells. Cells were transfected with or without 20 nM siIRF7 and then 24 h later infected with PulV at a moi 10 for a further 24 h. Virus containing supernatant was tested for PulV titre by TCID_50_. Experiments were performed in triplicate and results indicate mean values. Error bar represent standard error. The p-values were determined using two sample t-tests assuming unequal variances.

To explore the importance of bat IRF7 in the IFN production pathway, a knockdown approach was used in our *P. alecto* PaKiT03 cells. Transfection of siRNA targeting bat IRF7 (siIRF7) for 24 h resulted in a reduction of native IRF7 mRNA expression to approximately 20% compared to mock transfected cells. The statistical analysis of the knock down effects was calculated by comparing mRNA expression in the knock down samples to mock transfected cells. To exclude the possibility of off-target effects of siRNA transfection, the expression of a closely related gene, IRF3 was examined in transfected cells. As shown in Figure 4A, there was a decrease in IRF3 transcription in siIRF7 transfected cells due to possible toxic effects and/or off target effects of the siRNA smartpool but this change was not statistically significant. Having confirmed the knockdown effect of siIRF7, we then explored the downstream effect of reduced IRF7 on IFN-β production and viral replication. Two experiments were performed; firstly, 24 h after siIRF7 transfection, cells were stimulated with SeV for 6 h and IFN-β mRNA was detected by qPCR. Knockdown of IRF7 impaired the induction of IFN-β by SeV by 2.5 fold relative to untransfected cells (Figure 4B). Notably, bat cells maintained some IFN-β induction in siIRF7 cells, a result which likely reflects insufficient knockdown of IRF7 or IFN-β induction through alternative (IRF3 or NF-κB) pathways. To examine the effect of IFN knockdown on the replication of a bat-borne virus, PulV, a dsRNA reovirus originating from pteropid bats, was used to infect siIRF7-transfected bat cells [46]. A dose of 10 moi was used to infect PaKiT03 cells one day after siIRF7 transfection (or mock transfection). Cell supernatant containing virus was collected 24 h after infection and applied to a TCID_50_ test. Figure 4C shows that when bat IRF7 was knocked down, PulV replicated to a titer more than four-fold higher than in mock-transfected cells. These data demonstrate that bat IRF7 is functionally important in SeV induced IFN-β production and antiviral defense against PulV infection of bat cells.

### Bat IRF7 is activated by MyD88

**Figure 5:**
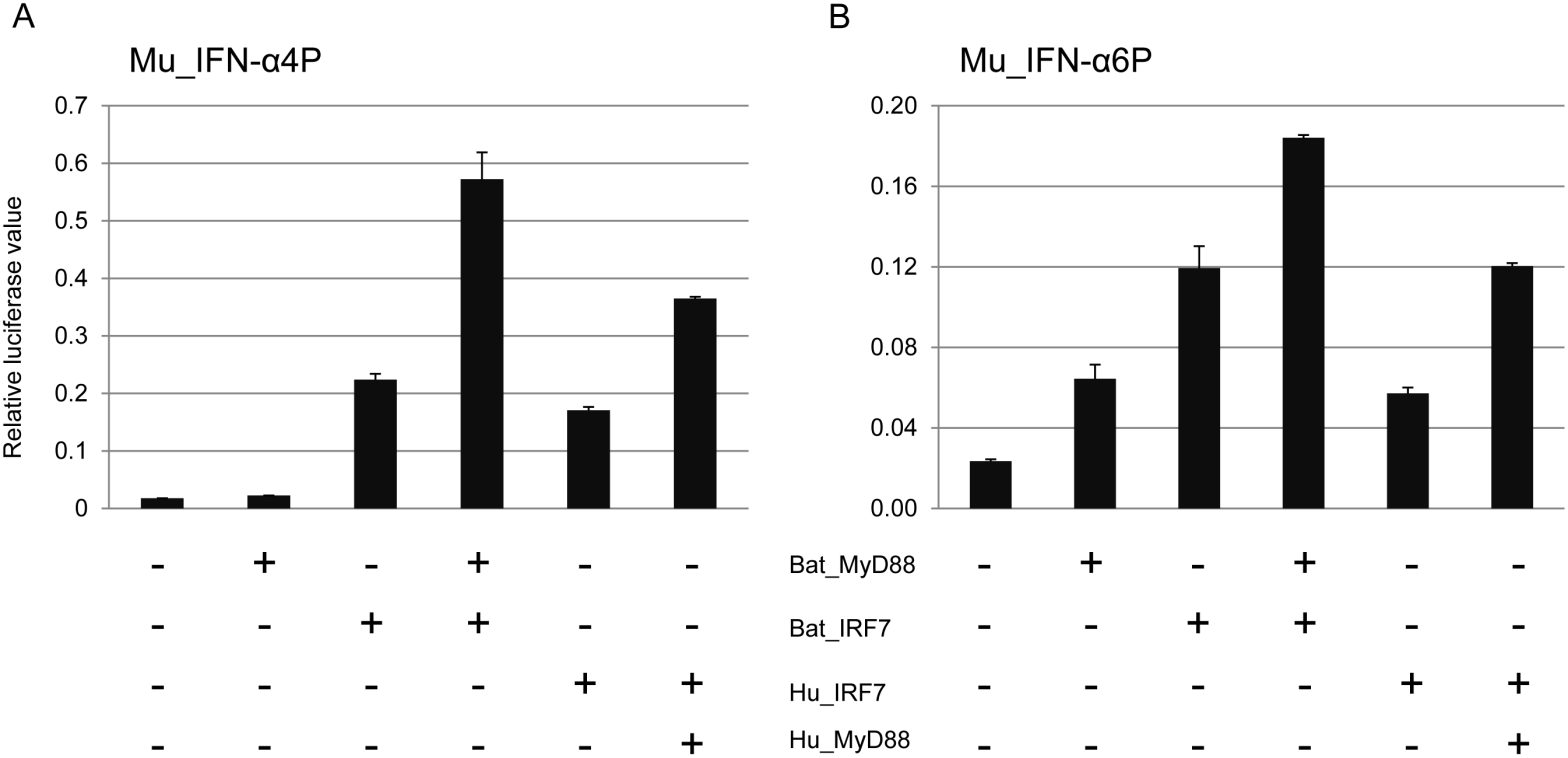
Bat IRF7 activation by bat MyD88. HEK293T cells were transiently co-transfected with bat MyD88 expression plasmid and mouse IFN-α4 (A) or IFN-α6 (B) promoter plasmid along with human IRF7, bat IRF7. After 30 h, cells were analysed for promoter activity by reporter gene luciferase assay. Data are mean values of two independent experiments and error bars represent standard errors.

We next wanted to determine whether bat IRF7 is involved in the production of IFN-α and IFN-β by the MyD88 despite the divergent nature of its MyD88 binding domain. The transactivation activity of bat IRF7 was compared to that of human IRF7 using expression plasmids containing bat or human MyD88 and IRF7 co-transfected with mouse IFN-α4 or IFN-α6 promoter plasmids. In mice, IFN-α4 is the earliest IFN-α induced by viral infection, while IFN-α6 is induced later in the response. A dose of 10 ng or 100 ng of IRF7 was co-transfected with MyD88 for the IFN-α4P and IFN-α6P promoter assay respectively in HEK293T cells. These doses of IRF7 were chosen as they do not result in huge induction of the native IFN promoter. As shown in Figure 5A, the activation of IFN-α4P by both human and bat IRF7 was increased by co-transfection with MyD88. Co-transfection of cells with bat MyD88 and IRF7 resulted in a higher response compared to co-transfection with the corresponding human plasmids. Our results demonstrate that even with a significant difference in its MyD88 binding region, bat IRF7 is still capable of inducing IFN-α transcription via MyD88 (Figure 5). Some differences were observed between IFN-α4 and IFN-α6 inducibility which may be due to differences in their IRF or NF-κB binding motifs. No IFN-α4 activation occurred following cotransfection of bat MyD88 with bat IRF3 confirming that IRF3 is MyD88 independent (Figure S5A). A similar experiment was performed to confirm that bat IRF3 is capable of activating the human IFN-β promoter confirming the activity of bat IRF3 (Figure S5B) [21]. Thus, only IFNA production by IRF7 is dependent on MyD88.

### Bat IRF7 interacts with bat MyD88

**Figure 6:**
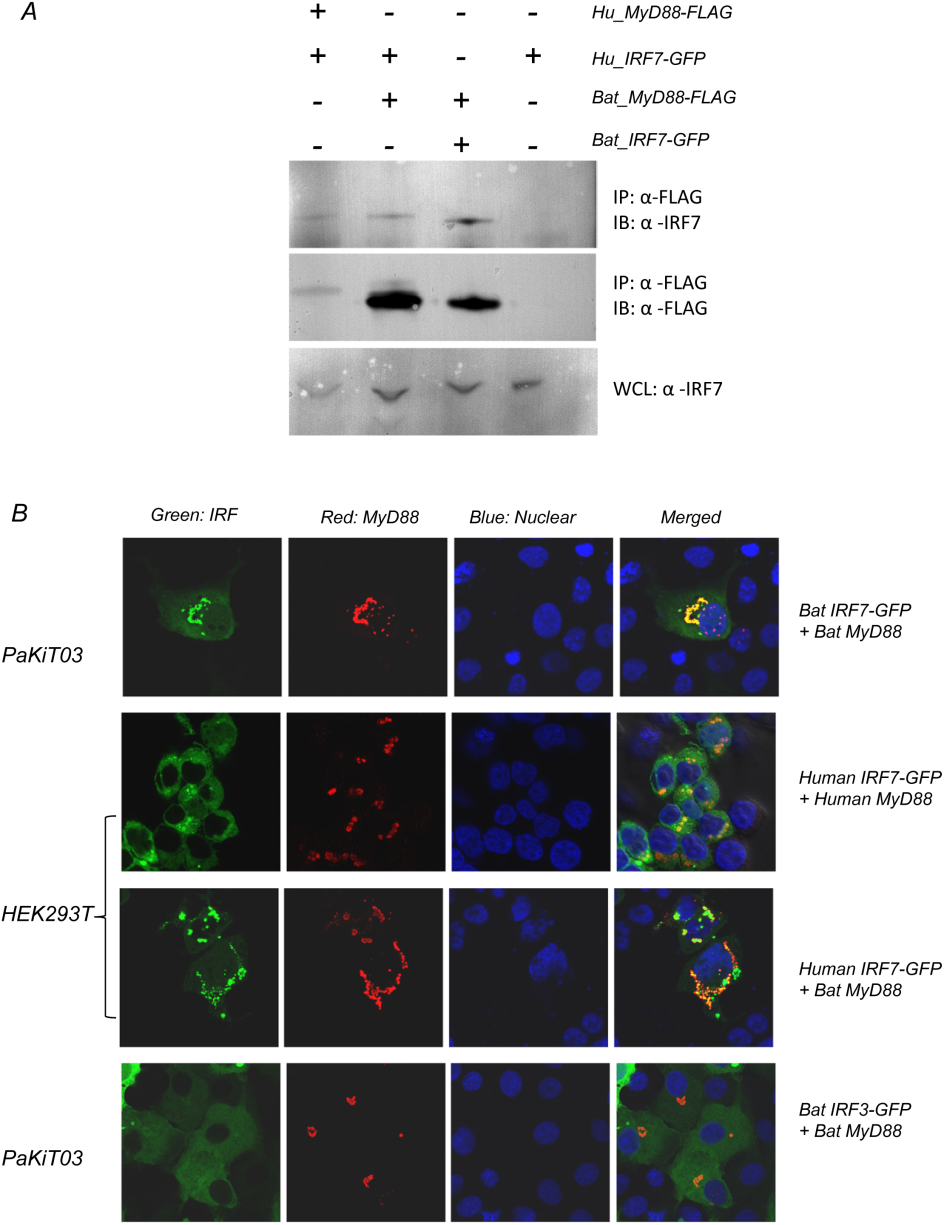
Bat IRF7 interacts with bat MyD88. (A) Binding of bat IRF7 to bat MyD88. HEK293T cells were transfected with GFP-tagged IRF7 and FLAG-tagged MyD88 (human or bat, as indicated). At 24 h post-transfection, whole cell lysate was prepared and immunoprecipitated with anti-FLAG antibody M2. Immunoprecipitated complexes (IP) were analysed by immunoblot for IRF7 and MyD88 expression using an anti-human IRF7 antibody (top panel), and an anti-FLAG antibody M2 for detection of FLAG tagged MyD88 (middle panel). Whole cell lysate (10 ul) was also run on an SDS-PAGE gel and subsequently analysed for IRF7 expression using anti-human IRF7 antibody (bottom panel). (B) Bat IRF7 co-localizes with bat MyD88. PaKiT03 cells were transfected with bat GFP-IRF7/IRF3 and bat FLAG-MyD88 and HEK293T cells were transfected with human GFP-IRF7 and human or bat FLAG-MyD88. 16 hours post-transfection (or 24 h), cells were fixed for indirect immunofluorescence assay using an anti-human MyD88 antibody and red fluorescence-conjugated secondary antibody. Pictures show co-localisation of bat IRF7 or bat IRF3 with bat MyD88 in PaKiT03 cells and human IRF7 or human IRF3 with human MyD88 in HEK293T cells.

To confirm that bat IRF7 interacts with bat MyD88, experiments were performed to examine the interaction between the two proteins. Firstly, HEK293T cells were co-transfected with plasmids encoding FLAG-tagged MyD88 (either human or bat, as indicated) and GFP-tagged IRF7 (human or bat). Cells were lysed and protein immunoprecipitated with anti-FLAG antibody conjugated beads followed by immunoblotting with anti-FLAG or anti-human IRF7 antibody. Human IRF7 protein was successfully captured by human MyD88 which was detected in IP samples demonstrating protein interaction between human MyD88 and human IRF7 (Figure 6A, panel 1). The detection of only a faint band may be due to the relatively low expression of the input human IRF7 and MyD88 proteins from these expression plasmids. Clear signals were detected for IRF7 and MyD88 following co-IP of bat MyD88 with bat or human IRF7 (Figure 6A). There is a slight difference in the molecular weights of human and bat MyD88 and IRF7 which are reflected on the blot (human and bat MyD88 are 33.3 and 33.6 KD respectively and human and bat IRF7 have a molecular weight of 54.2 and 55.6 KD respectively). These results clearly demonstrate that bat MyD88 protein is capable of binding both bat and human IRF7 proteins.

Confocal microscopy was used to determine the colocalisation of the two proteins, to further confirm protein interaction. Bat kidney PaKiT03 cells or human kidney HEK293T cells were used to examine colocalisation of IRF7 with MyD88. A dose of 200 ng/well of either human or bat MyD88 and IRF7 plasmids were used to transfect cells grown overnight on coverslips in 24-well plates. Sixteen hours later, cells were fixed and stained with anti-human MyD88 antibody and examined under the confocal microscope. MyD88 transfection alone resulted in the formation of very large condensed aggregates in the cytoplasm of both human and bat cells. Human MyD88 and human IRF7 colocalised in a manner similar to previous studies (Figure 6B) [21],[41]. Similarly, bat MyD88 and IRF7 proteins also demonstrated clear co-localisation. As shown in Figure 6B, bat IRF7 appeared to be surrounded by MyD88 in an aggregated form, which is the typical MyD88 structure [47],[48]. In addition, bat MyD88 also colocalised with human IRF7, which is consistent with our IP results (Figure 6A). As expected, no such aggregated structure was observed following co-expression of bat MyD88 with bat IRF3, ruling out the possibility of interaction between these two proteins.

### The virus activated domain of bat IRF7 is functionally conserved

**Figure 7:**
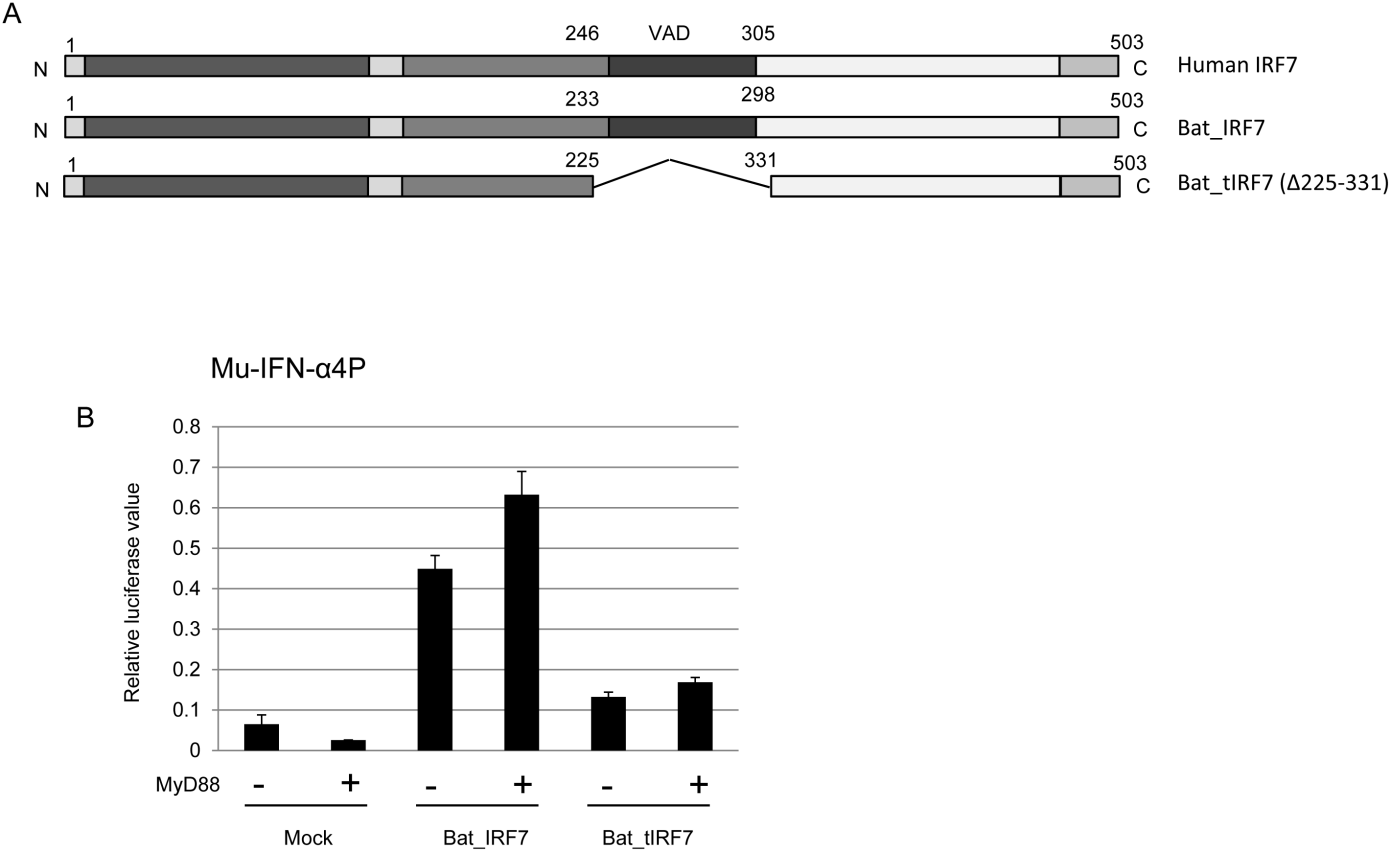
The VAD domain of bat IRF7 is functionally conserved with that of human IRF7. A. The full length human and bat IRF7 functional domain and a truncation mutant of bat IRF7 (Δ225–331, bat_tIRF7) are illustrated schematically. (B) Deletion of the VAD domain of bat IRF7 abolishes IFN transactivation. Bat IRF7 or bat tIRF7 plasmid was transfected into HEK293T cells with mouse IFN-α4 promoter plasmid with or without bat MyD88 expression plasmid and luciferase activity was tested 30 h post-transfection. Results show mean values of two experiments and error bars represent standard errors.

Having confirmed the functional reliance and binding capability of bat IRF7 to bat MyD88, it appeared that the poorly conserved MyD88-binding region (between aa 200–300) retains a similar function to the corresponding human domain. To further confirm this was the case, an internal deletion mutant of bat IRF7, tIRF7 was constructed (Figure 7A). This form of bat tIRF7 contained a deletion of aa 225–331 which has been shown to abolish the ability of human IRF7 to transactivate the IFN-α1 promoter [22]. The mouse IFN-α4 promoter transactivation activity by tIRF7 was compared to the full-length IRF7 protein using a luciferase assay. Having confirmed successful expression, Figure 7B clearly demonstrates that tIRF7, was unable to activate the mouse IFN promoter, even in the presence of MyD88 (Figure 7B). These data are consistent with functional conservation of the region corresponding to aa 233–298 in bats with that of human IRF7 despite significant sequence variation.

## Discussion

IRF7 is a master regulator of IFN expression in mammals and is therefore central to the innate antiviral immune response. In humans, IRF7 acts predominately in pDCs via activation of TLR7/9 and the MyD88 dependent signaling pathway [49]. Regulation of the IFN response may play an important role in the ability of bats to coexist with viruses in the absence of clinical signs of disease. This report describes the analysis of IRF7 from our model bat species, the Australian black flying fox, *P. alecto,* an important reservoir for viruses including Hendra virus, which has resulted in the deaths of numerous horses and humans since its discovery in 1994 [4],[46]. Our results support the conservation of functional activity of IRF7 in *P. alecto* but provide evidence of a wider tissue distribution which has implications for broader activation of the IFN response in bats. Our results provide the first functional characterization of IRF7 in any species of bat and contribute to our understanding of the function and evolution of IRF7 in mammals.

Bat IRF7 was identified from the bat genome and bat transcriptome data [39],[40], together with RT-PCR results from spleen cDNA resulting in the identification of a single full length variant of IRF7. In humans and mice, IRF7 expression is very low in most tissues and cells with the exception of pDCs and cells that have been activated by IFN [12],[15]. In contrast, the transcription of *P. alecto* IRF7 was detected not only in immune-related tissues but comparable expression was observed in many other organs as well. Although there is a lack of data on the tissue distribution of IRF7 in mammals besides human and mouse, there have been several studies on fish IRF7. Interestingly, at least five species of fish including crucian carp, mandarin fish, snakehead fish, Atlantic salmon and Japanese flounder express IRF7 constitutively in a wide variety of tissue types although different IRF7 transcripts were expressed in each species. These tissues were neither primarily immune-related nor serve as portals for microbial infection, where the immune response is easily initiated. Since these fish IRF7s can also be induced by dsRNA, they were hypothesised to play an important role in fish immunity [16],[17],[18],[19],[20]. Although further analysis of the cell types responsible for constitutive IRF7 expression in bats is required, a constitutively expressed IRF7 in a broad range of cells and organs may result in faster and stronger IFN production upon viral infection [49]. This observation is similar to the pattern of type III IFN receptor expression which has a wide distribution in bats but only limited distribution in other mammals [50]. Thus, bats may maintain the potential to rapidly activate the innate immune response in a broader subset of tissues and cells than other mammals.

Induction of IRF7 by treatment or transfection of our bat kidney cell line with the dsRNA ligand, polyI:C resulted in a peak in the induction of IRF7 at 9 h post-treatment, which is 3 h later than the peak in bat type I and type III IFNs but similar to that of ISGs Mx1, OAS1 and PKR described previously in bat cells [29],[33]. This result is consistent with the induction of IRF7 through type I IFN feedback similar to other species. In humans, IRF7 is generated through multiple pathways following IFN induction. Following the production of IFN and binding to the IFN-αR, a complex consisting of activated STAT1, STAT2 and IRF9, called the IFN stimulated gene factor 3 (ISGF3) is formed, which in turn binds to the ISRE on the IRF7 promoter and induces IRF7 transcription. The human IRF7 promoter region contains an NF-κB binding site and a single functional ISRE approximately 1.3-kb upstream from the ATG start site, both of which are important in the induction of IRF7 [49],[51]. Analysis of the putative bat IRF7 promoter region resulted in the identification of two ISREs and one NF-κB binding site indicating that multiple mechanisms for IRF7 activation may also exist in bats. However, two ISRE sites were also identified in the IRF7 promoters of other species examined (mouse, horse, cow, dog, cat and rat). Thus, whether the broad distribution of constitutively expressed IRF7 is the result of the presence of a more efficient IRF7 promoter region driven by transcription factors other than IRFs, or simply due to enriched immune-related cells in all tissues will require further study.

Sequence differences in the MyD88 binding domain of bat and human IRF7 led to the hypothesis that there may be functional differences in the activation of bat IRF7 and the regulation of the IFN response that may contribute to the ability of bats to resist the clinical outcomes of viral infection. Our results demonstrate that these sequences differences do not appear to affect IRF7 function either in IFN transactivation activity or activation by MyD88. Bat IRF7 was capable of activating both IFN-α and IFN-β promoters and the levels of transactivation were equivalent to or higher than that of human IRF7. Similarly, bat MyD88 and bat IRF7 maintained binding capability similar to their human counterparts. Deletion of the MyD88-binding region of bat IRF7 impaired its ability to activate IFN, demonstrating functional conservation of the MyD88 binding domain with that of human IRF7. Collectively, these data demonstrate that bat IRF7 is capable of inducing IFN and MyD88 binding in a similar manner to human IRF7.

Although the MyD88 binding domain of bat IRF7 has low sequence conservation with the equivalent region in human IRF7, experimental data demonstrate a fully functionally IRF7 exists in pteropid bats. Our results describing the experimental knockdown of IRF7 using siRNA is to our knowledge the first description of the use of siRNAs in bat cells. The successful knockdown of IRF7 is consistent with the presence of an RNA-silencing mechanism in bats similar to that in other mammals. IRF7 knockdown resulted in impaired IFN-β induction in SeV infected cells and enhanced PulV replication. Although further work will be required to determine whether IRF7 is the master regulator of the bat IFN response, these results confirm that IRF7 plays an important role in anti-viral defense and the early innate immune response in bats [13].

Analysis of the bat IFN-β promoter also revealed one residue difference in the PRDI module, known to be associated with activation by IRF3 or IRF7 [43]. A similar change in the human IFN-αP impairs its inducibility by IRFs [26]. Although bat IFN-β is strongly inducible in bat cells following either stimulation or viral infection, further work will be necessary to determine whether this mutation affects the induction of IFN-β under conditions other than those described here [29]. The presence of this mutation may also indicate that transcription factors other than IRF3 and IRF7 are involved in the regulation of IFNs in bats. Therefore, future work focusing on IFN promoters (including IFN-α and IFN-λ) will be necessary to explore whether the bat IRF-IFN induction pathway is as critical to IFN induction as it is in other species. In humans, there are around 1400 transcription factors that have been recognized [52]. Whether these factors play similar roles in bats or whether they perform different functions resulting in differences in the expression of downstream genes remains to be determined. Understanding the mechanisms responsible for the regulation of the IFN response will assist in discovering how bats successfully coexist with viruses.

In conclusion, our data clearly demonstrate that bat IRF7 exhibits a constitutive expression pattern across a broad range of immune and non-immune related organs, intact IFN transactivation function, and binds to and is activated by bat MyD88. These findings not only provide the first information on the MyD88-IRF7 dependent IFN production pathway in bats, but also explore the functionality of bat IRF3 and identify critical point mutations in the bat IFN-β promoter. Ultimately, we hope these advances may help uncover the mechanisms underlying the ability of bats to co-exist with deadly viruses, and that this will in turn lead to the development of potential therapeutic strategies targeting viral infections in other mammals.

## Supporting Information

Figure S1 **Phylogenetic analysis based on amino acid alignment of IRF7 from representative vertebrate species.** Branch support is indicated as the percentage of 1000 bootstrap replicates. Sequences are from the Ensembl database with the exception of *P. alecto* IRF7. *P. alecto* IRF7 is highlighted in bold.
(TIF)

Figure S2 **Alignment of the IRF7 DNA binding motif of** ***P. alecto*** **with other mammals.** Residues important for IRF7 binding to the IFN promoter (described in Genin et al, 2009) are highlighted in grey. Sequences from species other than *P. alecto* and *M. davidii* were obtained from the Ensembl database. Bat sequences from *P. alecto*, *P. vampyrus* and *M. davidii* genome are colored red.
(TIF)

Figure S3 **Alignment of MyD88 (in bold) from** ***P. alecto*** **with sequences from other mammals.** Residues which are identical to human MyD88 are shown as dashes while gaps are indicated by dots. The death domains which are responsible for activation of IRF7 proteins are boxed. Sequences from species other than *P. alecto* were obtained from the Ensembl database.
(TIF)

Figure S4 **Alignment of the** ***P. alecto*** **IFN-β promoter region with the corresponding region from human, horse, mouse, dog, pig and microbat.** If more than one IFN-β sequence was present, the gene identified as IFN-β1 was used in the analysis. Residues which are identical to the human sequence are shown as dashes while gaps are indicated by dots. Two modules named positive regulatory domain (PRD) III and I, responsible for binding to IRF3 and IRF7 in humans (Maniatis et al, 1998) are boxed. The TATA binding motif has also been boxed. The residue in the *P. alecto* IFN-β promoter which may have disabled PRDI has also been highlighted.
(TIF)

Figure S5 **Bat IRF3 can’t be activated by bat MyD88 (A) but can induce IFN-βP by itself (B).** HEK293T cells were transiently co-transfected with bat MyD88 expression plasmid and mouse IFN-α4 or Bat IFN-βP promoter plasmids along with bat IRF3. After 30 h, cells were analysed for promoter activity by reporter gene luciferase assay. Data are mean values of two independent experiments and error bars represent standard errors.
(TIF)
