# Supplementary figures and images for: IRF7 in the Australian Black Flying Fox, Pteropus alecto: Evidence for a Unique Expression Pattern and Functional Conservation
Source: PLoS One. 2014 Aug 6;9(8):e103875. doi: 10.1371/journal.pone.0103875 (PMC4123912; doi:10.1371/journal.pone.0103875)

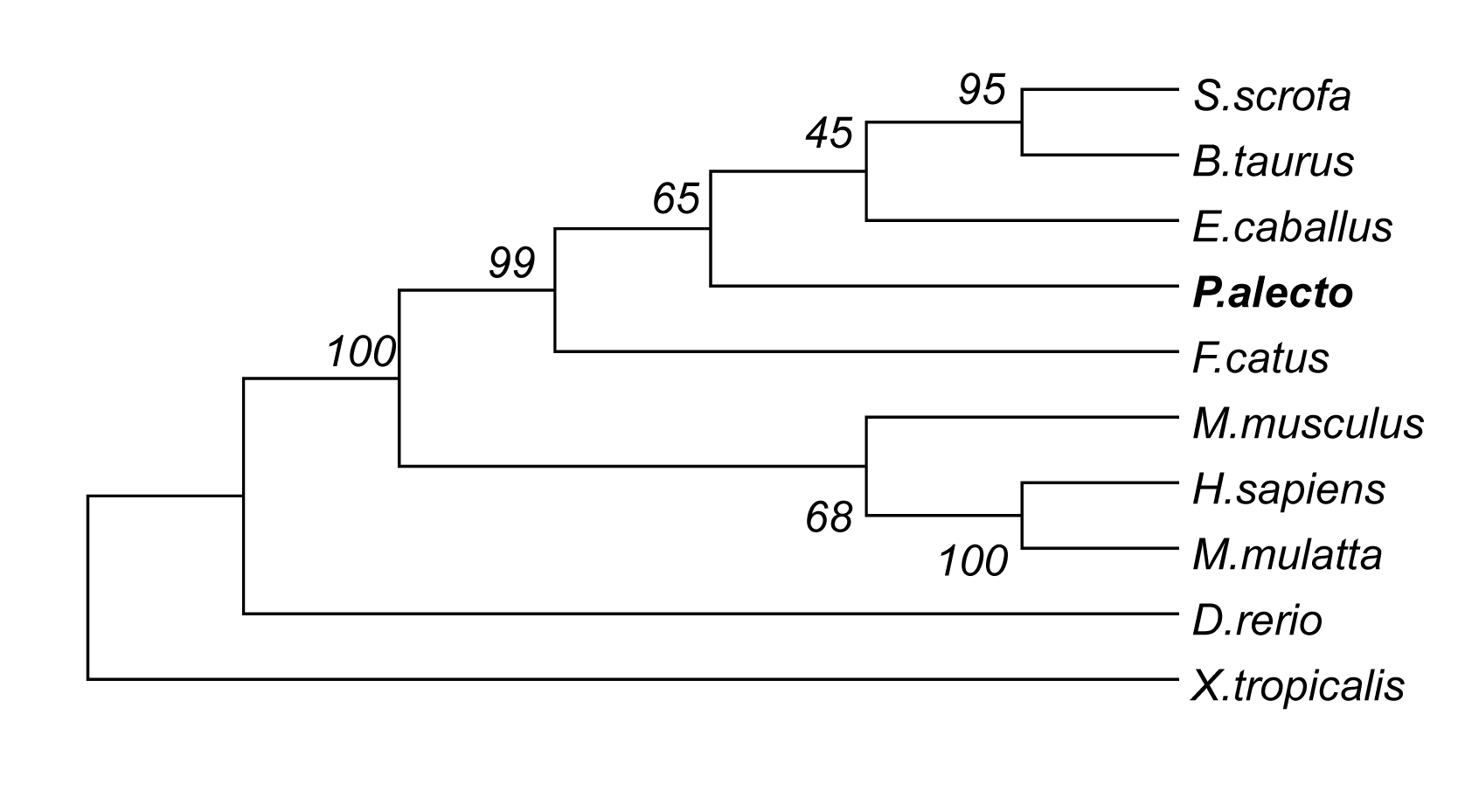

Supplement: Figure S1 — Phylogenetic analysis based on amino acid alignment of IRF7 from representative vertebrate species. Branch support is indicated as the percentage of 1000 bootstrap replicates. Sequences are from the Ensembl database with the exception of P. alecto IRF7. P. alecto IRF7 is highlighted in bold. (TIF) [file pone.0103875.s001.tif]

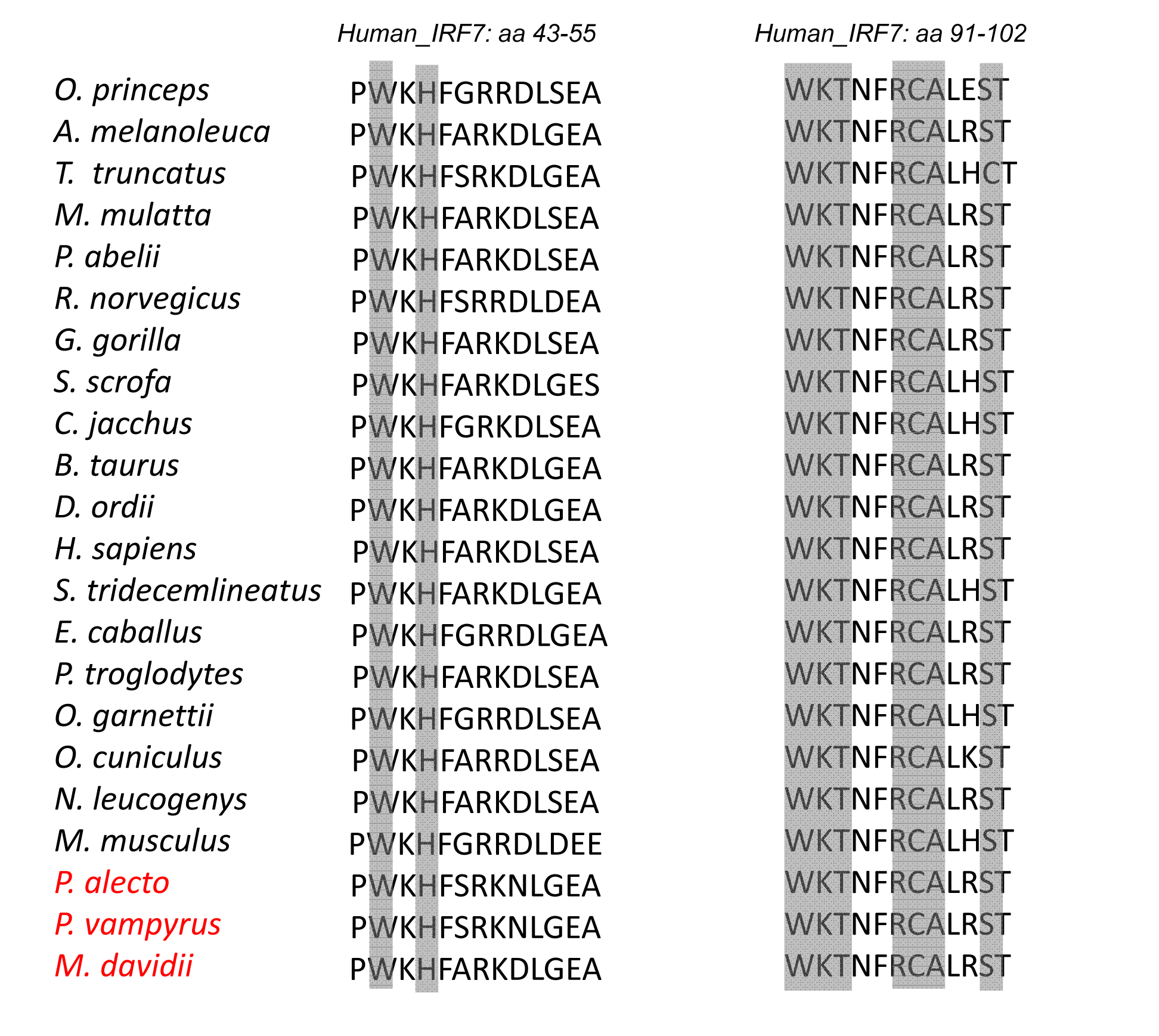

Supplement: Figure S2 — Alignment of the IRF7 DNA binding motif of P. alecto with other mammals. Residues important for IRF7 binding to the IFN promoter (described in Genin et al, 2009) are highlighted in grey. Sequences from species other than P. alecto and M. davidii were obtained from the Ensembl database. Bat sequences from P. alecto, P. vampyrus and M. davidii genome are colored red. (TIF) [file pone.0103875.s002.tif]

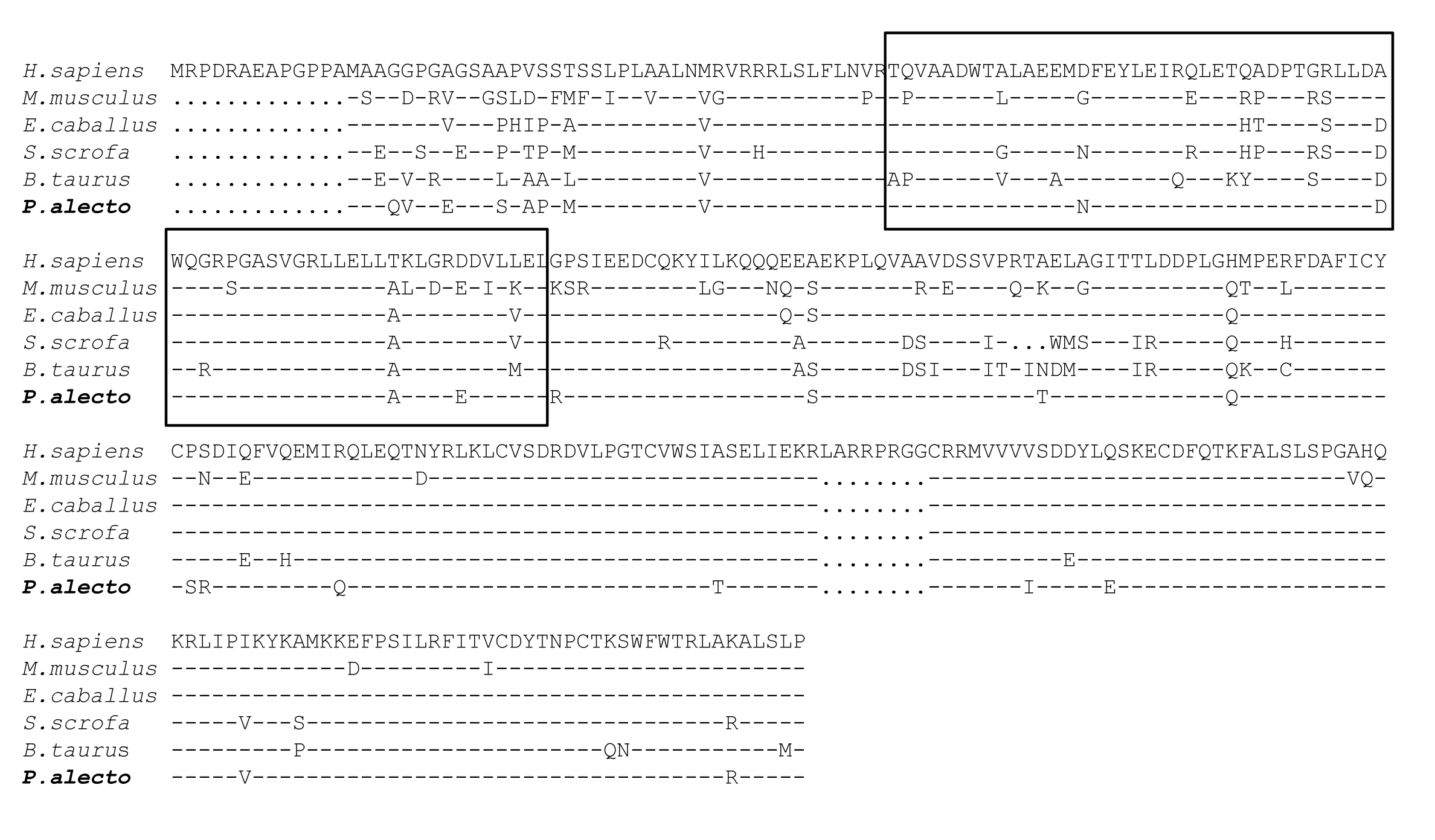

Supplement: Figure S3 — Alignment of MyD88 (in bold) from P. alecto with sequences from other mammals. Residues which are identical to human MyD88 are shown as dashes while gaps are indicated by dots. The death domains which are responsible for activation of IRF7 proteins are boxed. Sequences from species other than P. alecto were obtained from the Ensembl database. (TIF) [file pone.0103875.s003.tif]

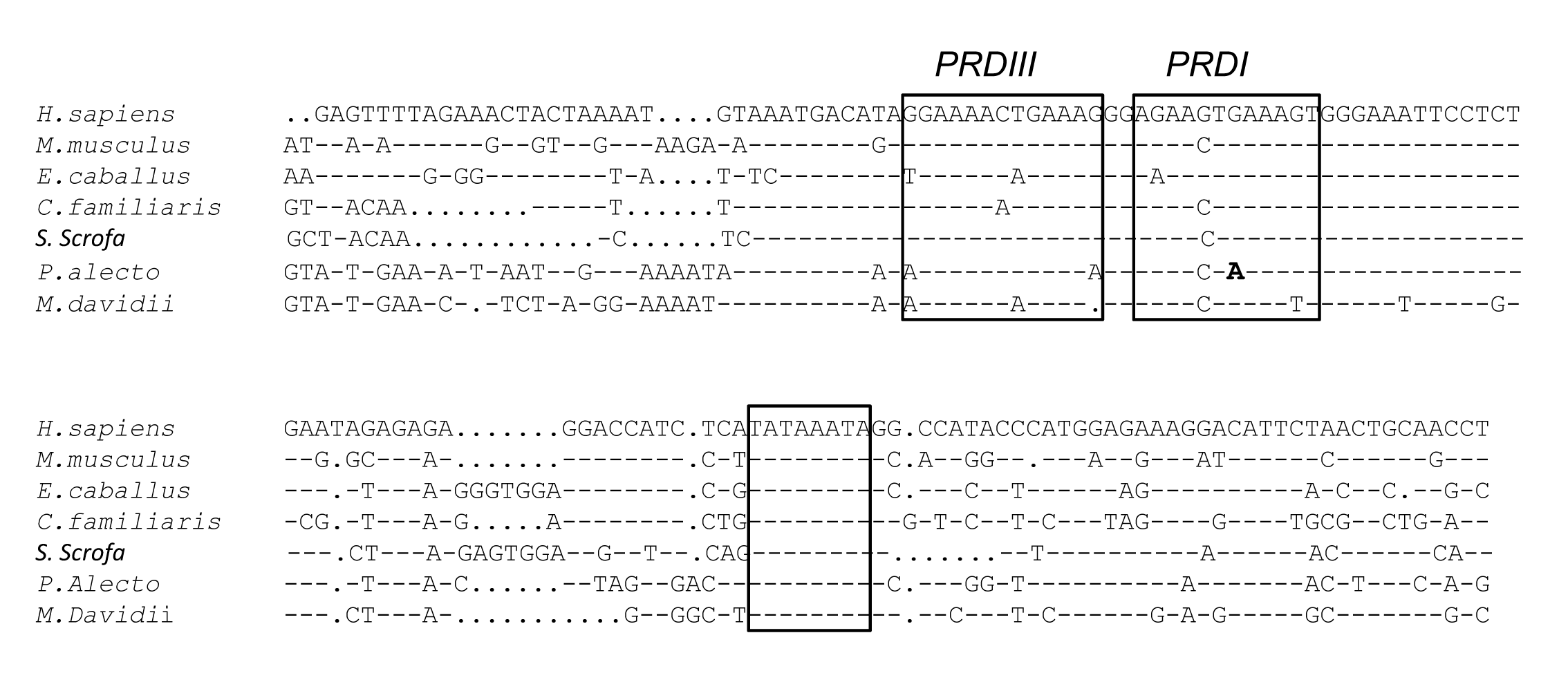

Supplement: Figure S4 — Alignment of the P. alecto IFN-β promoter region with the corresponding region from human, horse, mouse, dog, pig and microbat. If more than one IFN-β sequence was present, the gene identified as IFN-β1 was used in the analysis. Residues which are identical to the human sequence are shown as dashes while gaps are indicated by dots. Two modules named positive regulatory domain (PRD) III and I, responsible for binding to IRF3 and IRF7 in humans (Maniatis et al, 1998) are boxed. The TATA binding motif has also been boxed. The residue in the P. alecto IFN-β promoter which may have disabled PRDI has also been highlighted. (TIF) [file pone.0103875.s004.tif]

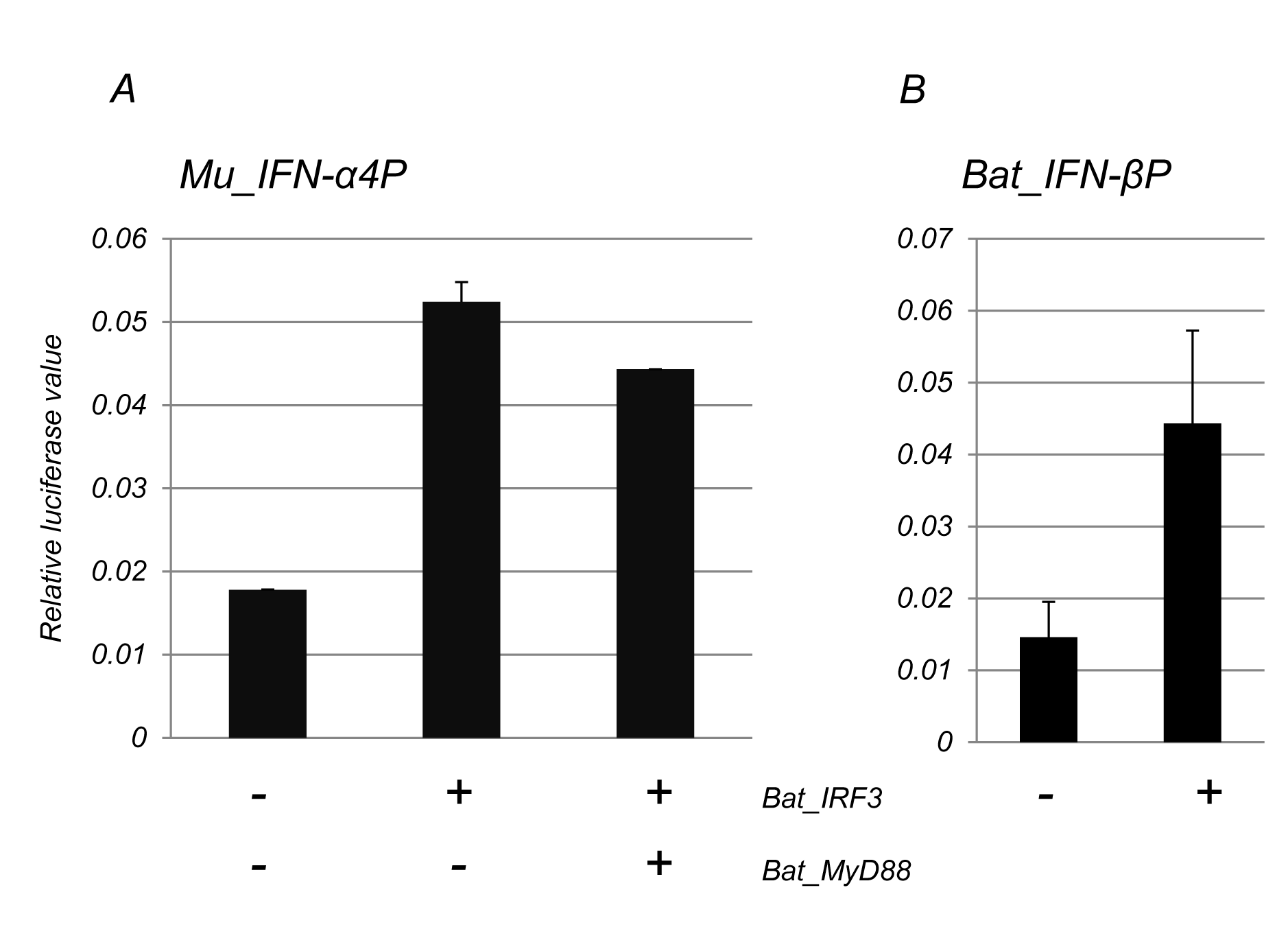

Supplement: Figure S5 — Bat IRF3 can’t be activated by bat MyD88 (A) but can induce IFN-βP by itself (B). HEK293T cells were transiently co-transfected with bat MyD88 expression plasmid and mouse IFN-α4 or Bat IFN-βP promoter plasmids along with bat IRF3. After 30 h, cells were analysed for promoter activity by reporter gene luciferase assay. Data are mean values of two independent experiments and error bars represent standard errors. (TIF) [file pone.0103875.s005.tif]
